# Perceptual Design and Evaluation of a Forearm-Based Vibrotactile Interface for Transfemoral Prosthetic Feedback

**DOI:** 10.3390/biomimetics11020112

**Published:** 2026-02-04

**Authors:** Mohammadmahdi Karimi, Sigurður Brynjólfsson, Kristín Briem, Árni Kristjánsson, Runar Unnthorsson

**Affiliations:** 1 School of Engineering and Natural Sciences, University of Iceland, 102 Reykjavík, Iceland; sb@hi.is (S.B.); runson@hi.is (R.U.); 2 School of Health Sciences, University of Iceland, 102 Reykjavík, Iceland; kbriem@hi.is (K.B.); ak@hi.is (Á.K.)

**Keywords:** proprioception, sensory substitution, tactile perception, transfemoral prosthesis, vibrotactile feedback, wearable haptics

## Abstract

The lack of reliable sensory input from prosthetic limbs limits transfemoral amputees’ ability to perceive limb movement without visual monitoring. This study evaluated design parameters of a proposed forearm-based vibrotactile system in a pre-clinical, design-level perceptual evaluation, conveying prosthetic joint positions through patterned vibrations to provide non-invasive proprioceptive feedback. Healthy participants completed two experiments assessing detection of tactile cues from dual-actuator bands on the wrist and elbow representing assumed ankle and knee positions. The effects of temporal structuring (sequential vs. simultaneous stimulation), actuator configuration, amplitude and frequency settings, and signal duration on response accuracy were examined. Sequential vibrations produced significantly higher recognition accuracy than simultaneous presentation (72.4% vs. 42.7%, *p* < 0.001) in a variety of vibration signal parameter values. Actuator placement also influenced performance: simultaneous stimulation on opposite forearm sides yielded significantly lower accuracy (*p* < 0.001) than same-side configurations, whereas this directional effect was not significant for sequential presentation. Accuracy did not differ significantly between equal and unequal amplitude or frequency levels across actuators. Longer stimulus durations improved accuracy, increasing from 82.3% at 60 ms to 92.5% at 240 ms, though the results indicated a saturation point, suggesting an optimal temporal window. These findings inform the design of forearm-based sensory feedback systems for improved prosthetic limb control.

## 1. Introduction

Modern transfemoral prostheses have steadily increased in mechanical sophistication in recent years, integrating motorized joints and adaptive control systems that allow for more natural and responsive movements [1,2]. However, despite these improvements, a critical limitation remains, which is the lack of intrinsic sensory feedback. Individuals with lower-limb amputations often struggle to perceive the position and movement of their prosthetic limbs, relying heavily on visual monitoring [3,4], and residual proprioception [5,6]. This reliance increases not only cognitive load but also reduces gait efficiency, increases the risk of falls, and negatively affects the user’s confidence in dynamic environments [7].

In response to these challenges, researchers have turned to sensory substitution systems that aim to restore some degree of proprioceptive awareness through alternative sensory channels [8,9,10]. Studies of lower limb prostheses have consistently emphasized the importance of sensory feedback in improving postural stability [11,12,13] and gait symmetry [14,15,16], reducing phantom limb pain [17], and enhancing the overall quality of life for amputees. Among the available modalities [18], vibrotactile feedback has shown promise due to its non-invasive nature, real-time responsiveness, and compatibility with wearable designs [19,20]. Vibrotactile systems can translate spatial or mechanical information into patterned vibrations applied to the skin, allowing users to perceive abstracted representations of movement or joint states [21,22].

To effectively implement such systems, it is essential to understand how different stimulus parameters influence users’ ability to accurately interpret these tactile signals. Conveying information through tactile stimulation requires careful design of feedback systems, including the actuators’ location on the body [23], the spatial arrangement of the actuators [24], the timing of signals [25], and the selection of the optimal vibration signal parameters [26,27,28]. Research on haptic interface design highlights the importance of aligning these factors with human perceptual capabilities to ensure that users can reliably distinguish between different stimuli.

We designed a forearm-based vibrotactile system to convey the assumed joint configurations of the knee and ankle of a transfemoral prosthesis. The forearm was chosen because it is a passive body part that does not interfere with other tasks and has been shown in previous research to offer good spatial acuity and reliable localization of vibrotactile stimuli, particularly near anatomical landmarks like the wrist and elbow [23,28]. Beyond perceptual acuity, forearm placement offers practical advantages for early-stage prototyping and future clinical translation, including independence from socket fit, reduced interference with locomotor mechanics, ease of donning and doffing, and compatibility with users who alternate between prosthetic devices. These considerations motivate the forearm as a promising site for cross-limb sensory substitution, particularly in exploratory design phases. The primary objective in the current research was to assess the perceptual accuracy of this feedback system across varying temporal and stimulus parameter conditions, including stimulation pattern presentation, signal duration, amplitude, frequency, and actuator location. By examining how these factors influence participants’ interpretation of tactile patterns associated with prosthetic configurations, the research results can contribute to the development of intuitive, non-invasive feedback systems for lower-limb prosthetics. The findings offer empirical insight into the best ways of designing efficient and user-friendly sensory substitution systems. By validating a feedback system that maps lower-limb prosthetic information to the forearm, the study introduces cross-limb mapping strategies with the potential to reduce users’ dependence on visual monitoring during ambulation and improve confidence in mobility.

## 2. Materials and Methods

### 2.1. Participants

This study included two laboratory experiments (Experiment 1 and Experiment 2). Ten healthy adults were recruited for Experiment 1 (5 males and 5 females, mean (SD), age 32.8 (5.1) years), while another set of 10 were recruited for Experiment 2 (7 males and 3 females, mean (SD), age 32.1 (6.2) years). Seven participants took part in both experiments, while the others were unique to one of the experiments. Experiment 1 and Experiment 2 were conducted on separate days. All participants provided informed consent prior to their participation. None of the participants reported any known neurological or tactile impairments and had either normal or corrected to normal vision. The study protocol was reviewed by the Icelandic National Bioethics Committee, which determined that formal ethical approval was not required (VSN2501016, 29 January 2025). The study was conducted in accordance with institutional ethical guidelines, and the declaration of Helsinki.

### 2.2. Apparatus

**Figure 1:**
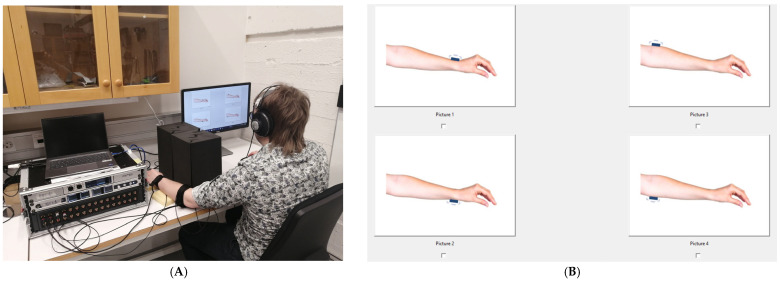
(**A**) The experimental setup, showing the wearable vibrotactile bands with actuators positioned on the forearm. (**B**) The response screen; observers clicked on the image depicting the site they thought had been stimulated (see Section 2).

The experimental setup and equipment involved a custom-built vibrotactile feedback system consisting of two wearable bands made of polyester webbing strap and hook-and-loop fasteners placed on the participant’s left forearm, one just above the wrist (distal) and the other just below the elbow (proximal). This placement was motivated by previous findings indicating that joint-adjacent locations yield significantly higher tactile discrimination accuracy than mid-forearm regions [28]. Each band contained two L5 (Lofelt GmbH, Berlin, Germany) voice-coil actuators enclosed in a 3D-printed housing to ensure safe and comfortable contact with the user’s skin, positioned to represent the ankle and knee joints, respectively. To simulate lower-limb joint positions using upper-limb tactile feedback, the vibrotactile actuators were used to encode four distinct positions of a transfemoral prosthesis. A full dorsiflexion of the ankle was encoded by activating the dorsal actuator on the wrist band, while full plantarflexion corresponded to activation of the volar wrist band actuator. Similarly, full knee extension was represented by activation of the dorsal actuator on the elbow band, and full knee flexion was encoded via the volar actuator on the elbow band. The choice to encode maximum flexion and extension of the ankle and knee was motivated by both perceptual and functional considerations. End-range joint positions are biomechanically salient, correspond to critical events during gait, and are often associated with stability, limb clearance, and load acceptance. From a sensory substitution perspective, these discrete states provide clear categorical boundaries, facilitating reliable perceptual discrimination during early-stage design evaluation. These mappings allowed users to interpret upper-limb tactile stimuli (on the forearm) as lower-limb joint positions. The vibrotactile feedback in this study was designed as an event-based cueing system rather than continuous joint-state feedback. Vibrotactile signals were delivered only when predefined joint positions were reached and were terminated immediately after signal delivery. No continuous stimulation was provided while the joint state was maintained. This design choice was made to isolate perceptual discrimination of discrete tactile events and to prevent sensory overload during multi-channel stimulation. Vibrotactile signals were generated by a computer running Python software (Version 3.12.3) and transmitted through an RME MADIface XT digital audio interface (RME GmbH, Chemnitz, Germany) and Ferrofish A32 digital-to-analog converters (Ferrofish GmbH, Linz am Rhein, Germany). Multi-channel amplifiers powered the actuators, and a graphical user interface (GUI) displayed on a computer screen allowed participants to interact with the system using a computer mouse (Figure 1).

### 2.3. Procedure

Upon arrival, each participant was briefed on the study’s purpose and procedures. The vibrotactile bands were then secured just above the wrist (distal) and just below the elbow (proximal). Participants were seated comfortably with their arm resting on a cushion (Figure 1A), and the system was adjusted to ensure consistent contact between the actuators and the skin. To eliminate any external auditory cues from the activation of the tactors, and isolate tactile perception, participants wore headphones playing white noise throughout the experiments. Additionally, a visual barrier was positioned between the participants and their stimulated forearm to prevent any visual feedback and, again, ensuring that observers had to solely rely on the tactile input.

To familiarize participants with the actuator layout, an introductory phase preceded the actual experiments in which single short pulses were delivered individually to each actuator. This phase was repeated as needed to ensure that participants could accurately associate each actuator with a specific joint location. Following familiarization in both experiments, participants started the main experimental session. During each trial, only one actuator on each band was activated: A vibrotactile pattern was delivered through the bands and participants were required to identify and reproduce the perceived pattern using the GUI (Figure 1B). Participants were given a 5-s window to respond after each stimulus presentation. If a participant responded before the time window expired, the system immediately proceeded to the next stimulus. If no response was made within the allotted time, the trial was automatically terminated, recorded as a missed response, and the experiment continued with the start of the next trial. At the end of the session, participants completed a questionnaire gathering their feedback, which was intended primarily to provide contextual information and assist with interpretation of the experimental results.

### 2.4. Experimental Parameters

The vibrotactile signals delivered to each actuator were sinusoidal waveforms. For each active channel, the vibration signal was computed using the following formula:

Signal = Amplitude × sin (2π × frequency (Hz) × t (s))(1)

where Amplitude is the normalized amplitude ranging from 0 (no vibration) to 1 (maximum vibration), corresponding to 0% to 100%, and t is the time vector defining the signal duration.

Although the vibrotactile stimuli represented assumed ankle and knee joint configurations, the stimulation parameters were not constrained by the biomechanics of walking. Instead, amplitude, frequency, duration, and temporal structuring were systematically varied to isolate their effects on tactile perception. This abstraction allowed controlled evaluation of perceptual performance independent of gait phase dynamics, which would otherwise confound interpretation of the effects of individual parameters.

#### 2.4.1. Experiment 1

The stimuli in the experiment were designed to primarily reveal the effect of temporal structuring on the accuracy of responses. In Experiment 1, the vibrotactile stimuli were delivered in two distinct presentation modes: simultaneous and sequential. In the simultaneous presentation mode, the actuators involved in generating the pattern were activated all at once, while in the sequential presentation mode, the actuator on the wrist band was always activated first, followed by the one on the elbow band, with a random interstimulus interval of either 10 ms or 100 ms. This fixed order was used to maintain a consistent distal-to-proximal mapping of the assumed ankle and knee joint information and the interstimulus interval manipulation was intended to minimize predictability and learning effects associated with fixed timing. The order of the two presentation modes was randomly alternated across participants, where half of them performed with the simultaneous mode first and the other half performed with the sequential mode first. A 5-min rest was provided between the two conditions.

The tested parameters included amplitude levels of 50% and 100% of the maximum possible intensity, stimulation frequencies of 125 Hz and 200 Hz, based on prior findings showing peak vibrotactile sensitivity on the wrist [26]. Across all conditions, there was a total of 64 unique stimulation states, involving all variations in the combinations of active actuator locations, amplitudes, and frequencies. Each combination was presented five times, resulting in a comprehensive test structure where all variables were randomized to minimize learning effects. Signal durations of 60 ms or 120 ms were randomly assigned across trials.

#### 2.4.2. Experiment 2

The purpose of Experiment 2 was to further investigate the effect of signal duration on perceptual discrimination. Only the sequential mode of vibrotactile presentation was used. The interstimulus interval was fixed at 100 ms, and four signal durations were tested: 60 ms, 120 ms, 180 ms, and 240 ms. Two amplitude levels (50% and 100%) and two frequency levels (125 Hz and 200 Hz) were also tested. The stimuli were presented in randomized order to prevent predictability. Each individual stimulation condition was presented only once, and no repetitions were included.

### 2.5. Data Collection and Analysis

In both experiments, data were collected automatically through a computer-controlled system that logged participant responses along with all relevant stimulus parameters, including actuator location, vibration amplitude and frequency, signal duration, and stimulation mode.

Analysis of variance (ANOVA) was conducted on the fitted models to evaluate the significance of main effects and interactions, using a significance threshold of α = 0.05. To handle repeated measurements from the same participants, we applied linear mixed-effects models in the ANOVA. In Experiment 1, the model included fixed effects of stimulation pattern, actuator location, amplitude consistency (equal or unequal across actuators), and frequency consistency (equal or unequal across actuators), with participant included as a random effect. In Experiment 2, a separate linear mixed-effects model assessed the effect of signal duration on recognition accuracy, also including participant as a random effect. Post hoc comparisons were performed using Tukey-adjusted tests to identify specific differences between conditions.

## 3. Results

### 3.1. Experiment 1

**Figure 2:**
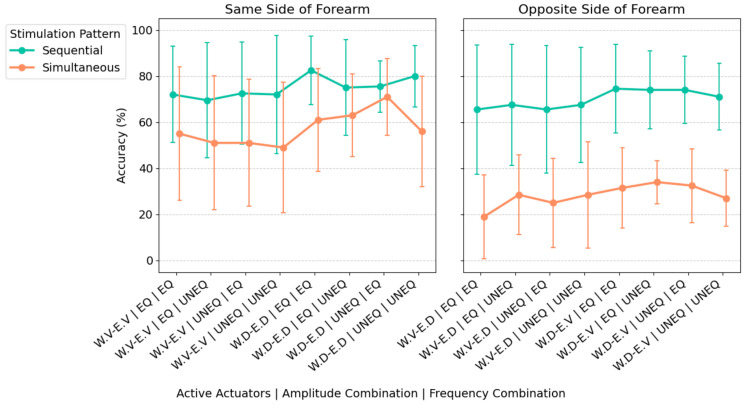
Recognition accuracy across simultaneous and sequential stimulation modes for the different actuator locations (W.V, Wrist. Volar; W.D, Wrist. Dorsal; E.V, Elbow. Volar; E.D, Elbow. Dorsal), amplitude, and frequency combinations (EQ, Equal; UNEQ, Unequal) on the same and opposite side of the forearm. Points indicate the mean recognition accuracy. Error bars represent ±1 standard deviation (SD).

The analysis revealed that sequential stimulation consistently produced significantly higher accuracy than simultaneous stimulation across nearly all actuator–amplitude–frequency combinations (Figure 2). For the sequential pattern, the mean recognition accuracy was 72.4%, while simultaneous stimulation showed greater variability with a mean accuracy of 42.7% (*p* < 0.001). A significant main effect of actuator location was found (*p* < 0.001), as well as a significant interaction between stimulation pattern and actuator location (*p* < 0.001). Post-hoc pairwise comparisons using Tukey-adjusted estimated marginal means revealed that opposing actuator directions within simultaneous stimulation negatively affected recognition accuracy. Specifically, when actuators in opposite locations (volar vs. dorsal part of forearm) were activated simultaneously, performance was significantly worse than when the two simultaneously activated ones were both on the volar or dorsal part of the forearm (*p* < 0.001). In contrast, there was no significant difference for the sequential pattern between the opposite and same direction of actuator locations (*p* = 0.437). No significant main effects or interactions were found for amplitude consistency (*p* = 0.867) or frequency consistency (*p* = 0.660).

### 3.2. Experiment 2

Although not part of the statistical analysis, data from Experiment 1 indicated that longer vibration signals tended to improve recognition accuracy. This raised the question of whether an optimal duration window might be identified. To explore the effect of extended signal duration on recognition accuracy, Experiment 2 was conducted. Based on the findings in Experiment 1, only the sequential presentation mode was used.

**Figure 3:**
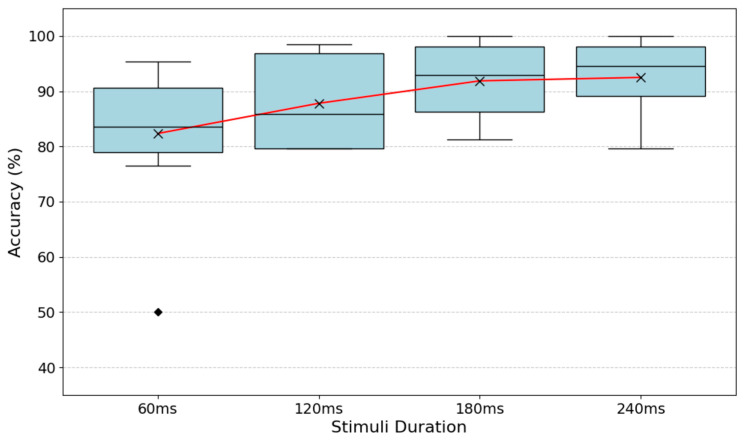
The results of experiment 2 showing the effect of vibrotactile signal duration on recognition accuracy during sequential presentation. Boxes show the interquartile range (IQR) with medians; whiskers represent 1.5× IQR, and diamond indicates outlier. ‘×’ shows mean accuracy, connected by a red line to illustrate the overall trend.

The results showed a significant effect of signal duration on accuracy (*p* < 0.001), with a clear increase in recognition accuracy as the duration of the vibrotactile signal increased (Figure 3). Post hoc Tukey-adjusted comparisons confirmed greater accuracy at 180 ms and 240 ms compared to the 60 ms stimulation (*p* = 0.003 and 0.0016, respectively). No difference was seen between accuracy of 120 ms, 180 ms, 240 ms, suggesting a ceiling effect.

## 4. Discussion

In this study the effectiveness of a wearable vibrotactile system, designed to encode transfemoral prosthetic joint configurations through upper-limb tactile feedback, was examined. The investigation focused on how temporal structuring, actuator configuration, differences in the amplitude and frequency of the actuator stimulation, and signal duration influence users’ ability to accurately identify stimulations. The findings reveal several important insights into tactile information processing and can have significant implications for the design of sensory substitution systems.

The first notable result was that sequential presentation of vibrotactile stimuli yielded far better recognition accuracy than simultaneous stimulation. It seems likely that this performance disparity can be attributed to the sensory system’s limited capacity for resolving concurrent tactile inputs. Sequential presentation, by temporally separating activation events, likely allows more precise encoding of each stimulus without interference from temporal overlap. This temporal clarity reduces perceptual ambiguity and increases the users’ ability to differentiate between complex tactile patterns. The findings were supported by participants’ questionnaire answers, where they reported greater difficulty discerning patterns during simultaneous vs. sequential stimulation, citing overlapping signals as the source of confusion. Several participants used expressions like “confusing,” or signals “blending together”. These results align well with those reported by Yeganeh et al. [25], who found that recognition accuracy during a tactile stimulation task was substantially higher for sequential (93.24%) than simultaneous (26.15%) presentation. The authors also attributed this performance difference to reduced interference and increased perceptual clarity. The differences in accuracy levels reported in the two studies may stem from methodological factors. In our experiment, shorter stimulus (60 ms or 120 ms vs. 500 ms) and interstimulus (10 ms or 100 ms vs. 450 ms) durations in the sequential conditions may constrain temporal encoding, negatively affecting signal recognition. Conversely, the simpler spatial configuration (2 × 2 vs. 2 × 3) and actuator placement in our simultaneous condition may have mitigated perceptual overlap, resulting in comparatively higher accuracy than reported by Yeganeh et al.

Notably, the locations of stimulations significantly only affected performance during simultaneous presentations. Combinations involving opposite directions (e.g., “Wrist Volar–Elbow Dorsal”) resulted in lower accuracy, which may disrupt spatial continuity and introduce ambiguity in the tactile signal. This likely impairs perceptual discrimination and increases the cognitive load required to differentiate between conflicting patterns. Conversely, the configurations allowing coherent and unidirectional mapping locations, such as “Wrist Volar–Elbow Volar”, produced better recognition rates, likely because they minimize perceptual interference by maintaining a congruent spatial direction along the forearm, which may facilitate more efficient encoding and signals discrimination. These findings underscore the importance of spatial mapping in vibrotactile interface design. The results indicate that intuitive actuator arrangements that reflect real-world prosthetic configurations facilitate quicker learning and improved perceptual accuracy.

Importantly, while the experimental protocol was designed to minimize short-term learning effects in order to isolate the influence of stimulation parameters, learning itself is an essential component of practical prosthetic use. In real-world scenarios, users are expected to progressively refine their interpretation of haptic feedback through repeated exposure and daily interaction with the system. Improvements in sensitivity, pattern recognition, and cognitive integration of vibrotactile cues are therefore indicators of system viability and usability. In this context, actuator configurations that promote intuitive spatial mapping are likely to support faster learning and more robust long-term adaptation.

Only slight differences in performance were observed when the amplitude and frequency differed versus when they were the same for both stimulations. However, further investigation is warranted before strong conclusions can be drawn from this. Although prior studies have reported reduced recognition accuracy during simultaneous vibrotactile stimulation, simultaneous presentation may be unavoidable in real-world prosthetic applications. We therefore hypothesized that introducing heterogeneous amplitude and frequency parameters across actuators could enhance perceptual separability and improve recognition performance under simultaneous stimulation. The present results indicate that this approach did not significantly increase accuracy, suggesting that parameter heterogeneity alone is insufficient to mitigate perceptual interference. Also, prior studies have shown that mismatches in amplitude can induce perceptual illusions such as the intensity order illusion [27], highlighting that incongruent stimulus parameters can distort tactile perception.

In Experiment 2, the focus was exclusively on sequential stimulation and the signal duration was systematically manipulated to determine how much exposure time is needed for participants to reliably recognize the vibrotactile patterns, essentially exploring the temporal limits of tactile encoding. The results showed a clear improvement in recognition accuracy with increased stimulus duration. This may reflect that shorter stimuli may not provide sufficient temporal information for full mechanoreceptor engagement, leading to degraded pattern resolution. In contrast, longer exposures facilitate better signal integration and cortical processing [29]. The observed plateau may suggest a point where enough perceptual information has been gathered, beyond which extending duration offers little additional benefit, a ceiling effect, in other words. Importantly, longer durations also resulted in more consistent performance across participants, indicating that temporal richness in the stimulus supports both accuracy and robustness. The finding that increased duration of sequential vibrotactile stimulation significantly increases recognition accuracy is consistent with the findings of Yeganeh et al. [25], which. underscores that optimizing stimulus duration is a critical parameter in the design of effective vibrotactile feedback systems. Participants, however, indicated in their feedback after the experiments that longer durations were sometimes harder to remember and interpret consistently. Additionally, users reported that extended signals felt more intense or overwhelming and therefore potentially uncomfortable on the skin, possibly due to the prolonged presence of vibrations at multiple locations.

A potential limitation of this study is that it involved only healthy participants rather than transfemoral amputees. Although lower-limb amputation does not directly affect forearm cutaneous sensation, differences in body representation [30,31] and multisensory integration [32,33] have been reported in amputee populations due to cortical reorganization, phantom limb phenomena, and compensatory strategies which may influence how artificial sensory feedback is cognitively interpreted. Nevertheless, the use of able-bodied participants is a common and necessary step in early-stage development of sensory feedback systems, particularly given the relatively small and heterogeneous amputee population. Initial validation in healthy participants provides valuable insight into perceptual limits and parameter optimization before clinical translation. The sample size might also be a limitation, and the partial overlap in participants across experiments. But note that this sample size is relatively similar to the sample sizes previously used in similar studies and that the statistical tests have low associated *p*-values, indicating considerable statistical power for answering our key questions. Although randomization, counterbalancing, and rest intervals were used to reduce learning effects, carryover across experiments cannot be fully excluded. It should be noted that proprioception is a multidimensional sense influenced by position, movement, velocity, and acceleration. The vibrotactile feedback investigated here encodes discrete spatial and temporal patterns rather than continuous kinematic variables. While this limits the representation of dynamic movement properties such as speed perception, the approach is well suited for conveying state-based information relevant to gait phase awareness. Additionally, the study was conducted in a seated, distraction-free laboratory setting, which does not reflect the complexities of real-world prosthesis use, where users must interpret sensory input while walking [34], navigating environments, and managing competing attentional demands. Accordingly, the present findings should be interpreted as design-level guidance for forearm-based vibrotactile encoding rather than evidence of functional benefits during ambulation. Integration with prosthesis-embedded sensors and validation during walking tasks constitute essential next steps, which are enabled but not addressed by the perceptual constraints identified here.

## 5. Conclusions

In this study a forearm-based vibrotactile feedback system was tested, with the future intent of conveying information on prosthetic joint positions and thereby proprioceptive feedback for transfemoral prosthesis users. Sequential stimulation significantly outperformed simultaneous stimulation, most likely due to reduced sensory interference. Spatially intuitive actuator placements and matched signal properties also contributed to improved recognition accuracy. Increasing stimulus duration improved performance up to a saturation point, close to 100%, indicating an optimal window for tactile encoding. These findings highlight key design considerations such as temporal structuring, spatial mapping, stimulation signal parameters, and duration optimization for developing intuitive, non-invasive sensory feedback systems. The validated cross-limb mapping approach offers promising potential for improving prosthesis control and user confidence in real-world settings.

## Data Availability

Dataset available on request from the authors.

## Abbreviations

The following abbreviations are used in this manuscript:

Ms	Millisecond(s)
SD	Standard deviation
GUI	Graphical user interface
Hz	Hertz (frequency unit)
Sec	Second
ANOVA	Analysis of Variance
W.V	Wrist. Volar
W.D	Wrist. Dorsal
E.V	Elbow. Volar
E.D	Elbow. Dorsal
EQ	Equal
UNEQ	Unequal
IQR	Interquartile range
